# Aqua­(2,2′-bipyridine-κ^2^ *N*,*N*′)(2-methyl­malonato-κ^2^ *O* ^1^,*O* ^3^)copper(II) dihydrate

**DOI:** 10.1107/S1600536812024889

**Published:** 2012-06-13

**Authors:** P. Manochitra, N. Manikandan, S. Murugavel, R. Sreeshailam, P. Sambasiva Rao

**Affiliations:** a Department of Chemistry, Pondicherry University, Puducherry 605 014, India; b Department of Physics, Bharathidasan Engineering College, Nattrampalli, Vellore 635 854, India; c Department of Physics, Thanthai Periyar Government Institute of Technology, Vellore 632 002, India

## Abstract

In the title compound, [Cu(C_4_H_4_O_4_)(C_10_H_8_N_2_)(H_2_O)]·2H_2_O, the Cu^II^ ion displays a slightly distorted square-pyramidal coordination. The water mol­ecule at the apical position shows a long bond [Cu—O = 2.276 (2) Å]. The basal plane is formed by two N atoms of the 2,2′-bipyridine ligand and two carboxyl­ate O atoms from a malonate group. The five-membered chelate ring is almost planar [maximum deviation = −0.006 (2) Å], while the six-membered chelate ring defined by the malonate ligand adopts a distorted boat conformation. In the crystal, Cu^II^ complex mol­ecules and lattice water mol­ecules are connected by O—H⋯O and C—H⋯O hydrogen bonds. The crystal packing is further stabilized by π–π inter­actions [centroid–centroid distances = 3.563 (2)–3.828 (2) Å].

## Related literature

For background to the applications of copper(II)–malonate complexes, see: Braga *et al.* (1998 ▶); Suresh & Bhadbhade (1997 ▶). For related structures, see: Gasque *et al.* (1998 ▶); Cui *et al.* (2005 ▶). For ring puckering analysis, see: Cremer & Pople (1975 ▶).
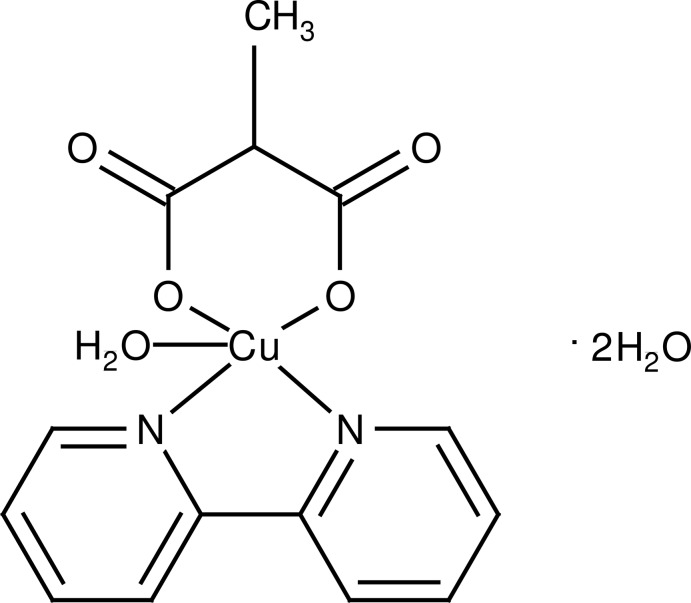


## Experimental

### 

#### Crystal data

[Cu(C_4_H_4_O_4_)(C_10_H_8_N_2_)(H_2_O)]·2H_2_O
*M* *_r_* = 389.84
Monoclinic, 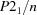

*a* = 10.7588 (7) Å
*b* = 7.4761 (6) Å
*c* = 20.1029 (13) Å
β = 90.917 (6)°
*V* = 1616.7 (2) Å^3^
*Z* = 4
Mo *K*α radiation
μ = 1.39 mm^−1^
*T* = 293 K
0.25 × 0.23 × 0.17 mm

#### Data collection

Oxford Diffraction Xcalibur Eos diffractometer
Absorption correction: multi-scan (*CrysAlis PRO*; Oxford Diffraction, 2009 ▶) *T*_min_ = 0.699, *T*_max_ = 0.790
9120 measured reflections
3782 independent reflections
2771 reflections with *I* > 2σ(*I*)
*R*_int_ = 0.045

#### Refinement

*R*[*F* ^2^ > 2σ(*F* ^2^)] = 0.049
*wR*(*F* ^2^) = 0.119
*S* = 1.05
3782 reflections
242 parameters
6 restraints
H atoms treated by a mixture of independent and constrained refinement
Δρ_max_ = 0.97 e Å^−3^
Δρ_min_ = −0.52 e Å^−3^

### 

Data collection: *CrysAlis CCD* (Oxford Diffraction, 2009 ▶); cell refinement: *CrysAlis RED* (Oxford Diffraction, 2009 ▶); data reduction: *CrysAlis RED*; program(s) used to solve structure: *SHELXS97* (Sheldrick, 2008 ▶); program(s) used to refine structure: *SHELXL97* (Sheldrick, 2008 ▶); molecular graphics: *ORTEP-3* (Farrugia, 1997 ▶); software used to prepare material for publication: *SHELXL97* and *PLATON* (Spek, 2009 ▶).

## Supplementary Material

Crystal structure: contains datablock(s) global, I. DOI: 10.1107/S1600536812024889/bt5931sup1.cif

Structure factors: contains datablock(s) I. DOI: 10.1107/S1600536812024889/bt5931Isup2.hkl

Additional supplementary materials:  crystallographic information; 3D view; checkCIF report

## Figures and Tables

**Table 1:** Hydrogen-bond geometry (Å, °)

*D*—H⋯*A*	*D*—H	H⋯*A*	*D*⋯*A*	*D*—H⋯*A*
C4—H4⋯O3^i^	0.93	2.59	3.457 (4)	155
O3—H3*A*⋯O4^ii^	0.82 (3)	1.97 (4)	2.775 (3)	170 (3)
O3—H3*B*⋯O6^iii^	0.85 (5)	1.90 (5)	2.744 (4)	173 (4)
O6—H6*A*⋯O5^iv^	0.84 (2)	1.96 (2)	2.787 (3)	168 (4)
O6—H6*B*⋯O5^v^	0.84 (1)	1.97 (1)	2.800 (4)	170 (4)
O7—H7*A*⋯O4^iv^	0.83 (1)	2.12 (2)	2.907 (4)	158 (4)
O7—H7*B*⋯O6^vi^	0.84 (1)	2.10 (1)	2.932 (5)	170 (4)
C2—H2⋯O7^vii^	0.93	2.50	3.256 (5)	139
C12—H12⋯O4^ii^	0.98	2.47	3.300 (5)	142

Symmetry codes: (i) 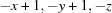; (ii) 
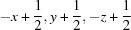
; (iii) 
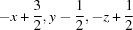
; (iv) 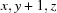; (v) 
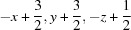
; (vi) 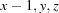; (vii) 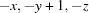.

**Fig. 1.**
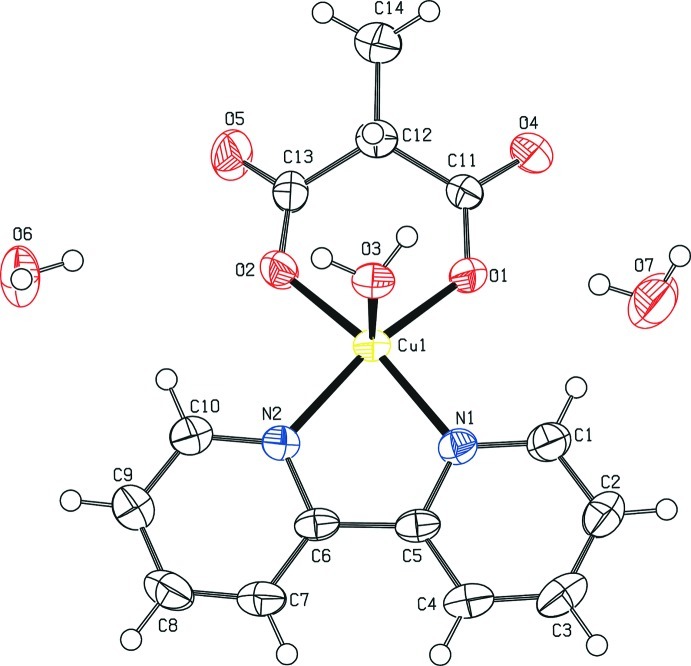
The molecular structure of the title compound with the atom numbering scheme. Displacement ellipsoids are drawn at the 20% probability level. H atoms are presented as a small cycles of arbitrary radius.

**Fig. 2.**
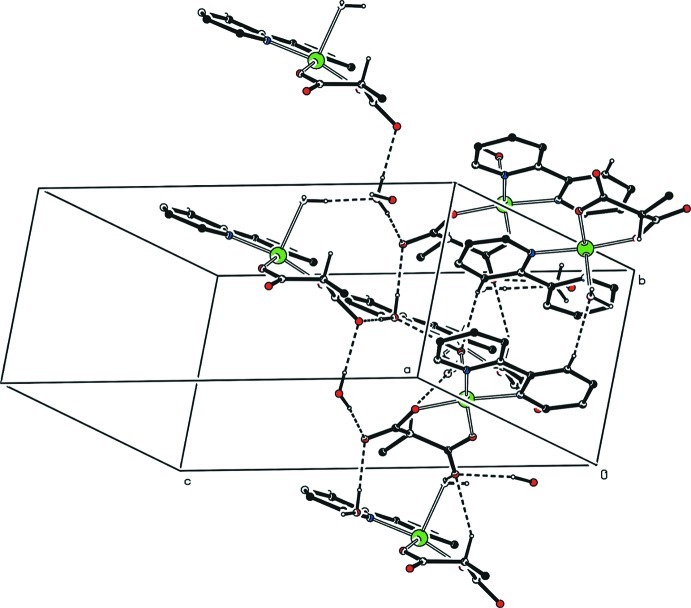
Part of the crystal structure showing O—H···O and C—H···O hydrogen bonds.

## supplementary crystallographic information

### Comment

The copper(II)—malonate complexes with suitable N-heterocyclic auxiliary ligands are of interest because the metal-N-heterocyclic chelate ring could influence the Cu—O(carboxyl) bond lengths and exhibits some degree of 'metalloaromaticity' (Suresh & Bhadbhade, 1997). On the other hand, self-assembly processes involving metal ions and organic ligands has attracted increasing attention for the development of novel functional materials with desired properties (Braga *et al.*, 1998). In continuation of the structural studies of metal complexes of these ligands, the crystal structure of the title compound was determined.

Fig. 1. shows a displacement ellipsoid plot of the title complex. The Cu^II^ion displays a slightly distorted quadratic pyramidal geometry and is coordinated to two N atoms of a 2,2'-bipyridine ligand and two carboxylate O atoms from a malonate group in the basal plane, and to a water molecule in the apical position [Cu1–O3 = 2.276 (2) Å]. The Cu1^II^ ion is displaced by -0.2382 (4) Å from the basal plane (N1/N2/O1/O2) towards the apical position. The O3 atom of the water molecule coordinated in the apical position deviates from this basal plane by 2.514 (2) Å. A similar coordination behaviour is observed in a similar structure (Gasque *et al.*, 1998), in which Cu1 deviates by 0.239 (2) Å and O3 atom by 2.533 (3) Å from the corresponding basal plane. The angle subtended by the pyridine ligand at the metal atom is far from the ideal value of 90° [81.0 (1)° for N1—Cu1—N2]. The bond distances Cu1—N1 = 2.004 (3), Cu1—N2 = 2.001 (1), Cu1—O1 = 1.907 (2) and Cu1—O2 = 1.919 (2) Å agree well with those reported for similar structures (Gasque *et al.*, 1998; Cui *et al.*, 2005).

The five-membered chelate ring (N1/N2/C5/C6/Cu1) is almost planar [maximum deviation = -0.006 (2) Å for atom N1], while the six-membered chelate ring defined by the malonate ligand (O1/O2/C11/C12/C13/Cu1) adopts a slightly distorted boat conformation as indicated by the puckering parameters (Cremer & Pople, 1975): Q = 0.580 (3) Å, θ = 81.8 (3)° and φ = 187.9 (3)°.

The crystal packing is stabilized by extensive intermolecular O—H···O and C—H···O hydrogen bonding interactions (Table 1) between the copper complex and uncordinated water molecules (Fig. 2). The crystal packing is further stabilized by π—π interactions with *Cg*1—*Cg*1^viii^, *Cg*1—*Cg*3^viii^, *Cg*3—*Cg*1^viii^, *Cg*3—*Cg*4^viii^, *Cg*4—*Cg*3^viii^, *Cg*3—*Cg*4^i^ and *Cg*4—*Cg*3^i^ seperations of 3.563 (2), 3.828 (2), 3.828 (2), 3.805 (2), 3.805 (2) 3.720 (2) and 3.720 (2) Å (*Cg*1, *Cg*2, *Cg*3 and *Cg*4 are the centroids of Cu1/N1/N2/C5/C6 ring, Cu1/O1/O2/C11/C12/C13 ring, N1/C1–C5 pyridine ring and N2/C6–C10 pyridine ring, respectively, symmetry codes: (i) 1-*x*, 1-*y*, -*z*; (viii) 1-*x*, -*y*, -*z*).

### Experimental

Basic copper(II) carbonate (1 mmol) was treated with an aqueous solution (10 ml) of methylmalonic acid (2 mmol) in a steam bath until the solid disappeared. The solution was then filtered and diluted to approximately 40 ml with water. An ethanol solution (10 ml) of 2,2'-bipyridine (2 mmol) was then added to above solution. The resultant clear-blue solution was warmed on a steam bath for 1 h. The volume was kept constant by periodic addition of water. Then the solution was filtered and allowed to stand at room temperature. Blue single crystals were obtained after 2 days. They were filtered, washed with water, ethanol and air dried.

### Refinement

H atoms of the water molecules were located in a difference fourier map, and were refined with distance restraints of O—H = 0.84 (1) Å and H···H = 1.32 (1) Å. All other H atoms were positioned geometrically, with C—H = 0.93–0.98 Å and constrained to ride on their parent atom, with *U*_iso_(H)=1.5*U*_eq_ for methyl H atoms and 1.2*U*_eq_(C) for other H atoms.

### Crystal data

**Table:**

[Cu(C_4_H_4_O_4_)(C_10_H_8_N_2_)(H_2_O)]·2H_2_O	*F*(000) = 804
*M**_r_* = 389.84	*D*_x_ = 1.602 Mg m^−^^3^
Monoclinic, *P*2_1_/*n*	Mo *K*α radiation, λ = 0.71073 Å
Hall symbol: -P 2yn	Cell parameters from 4366 reflections
*a* = 10.7588 (7) Å	θ = 2.9–29.2°
*b* = 7.4761 (6) Å	µ = 1.39 mm^−^^1^
*c* = 20.1029 (13) Å	*T* = 293 K
β = 90.917 (6)°	Plate, blue
*V* = 1616.7 (2) Å^3^	0.25 × 0.23 × 0.17 mm
*Z* = 4	

### Data collection

**Table:**

Oxford Diffraction Xcalibur Eos diffractometer	3782 independent reflections
Radiation source: fine-focus sealed tube	2771 reflections with *I* > 2σ(*I*)
Graphite monochromator	*R*_int_ = 0.045
Detector resolution: 15.9821 pixels mm^-1^	θ_max_ = 29.2°, θ_min_ = 2.9°
ω scans	*h* = −14→13
Absorption correction: multi-scan (*CrysAlis PRO*; Oxford Diffraction, 2009)	*k* = −10→9
*T*_min_ = 0.699, *T*_max_ = 0.790	*l* = −25→26
9120 measured reflections	

### Refinement

**Table:**

Refinement on *F*^2^	Primary atom site location: structure-invariant direct methods
Least-squares matrix: full	Secondary atom site location: difference Fourier map
*R*[*F*^2^ > 2σ(*F*^2^)] = 0.049	Hydrogen site location: inferred from neighbouring sites
*wR*(*F*^2^) = 0.119	H atoms treated by a mixture of independent and constrained refinement
*S* = 1.05	*w* = 1/[σ^2^(*F*_o_^2^) + (0.0472*P*)^2^ + 0.4525*P*] where *P* = (*F*_o_^2^ + 2*F*_c_^2^)/3
3782 reflections	(Δ/σ)_max_ < 0.001
242 parameters	Δρ_max_ = 0.97 e Å^−^^3^
6 restraints	Δρ_min_ = −0.52 e Å^−^^3^

### Special details

**Table:**

Geometry. All e.s.d.'s (except the e.s.d. in the dihedral angle between two l.s. planes) are estimated using the full covariance matrix. The cell e.s.d.'s are taken into account individually in the estimation of e.s.d.'s in distances, angles and torsion angles; correlations between e.s.d.'s in cell parameters are only used when they are defined by crystal symmetry. An approximate (isotropic) treatment of cell e.s.d.'s is used for estimating e.s.d.'s involving l.s. planes.
Refinement. Refinement of *F*^2^ against ALL reflections. The weighted *R*-factor *wR* and goodness of fit *S* are based on *F*^2^, conventional *R*-factors *R* are based on *F*, with *F* set to zero for negative *F*^2^. The threshold expression of *F*^2^ > σ(*F*^2^) is used only for calculating *R*-factors(gt) *etc*. and is not relevant to the choice of reflections for refinement. *R*-factors based on *F*^2^ are statistically about twice as large as those based on *F*, and *R*- factors based on ALL data will be even larger.

### Fractional atomic coordinates and isotropic or equivalent isotropic displacement parameters (Å^2^)

**Table:**

	*x*	*y*	*z*	*U*_iso_*/*U*_eq_	
H6A	0.8000 (17)	0.941 (4)	0.244 (2)	0.084 (16)*	
H6B	0.863 (3)	1.0935 (14)	0.242 (2)	0.078 (17)*	
H7A	0.0996 (16)	0.899 (6)	0.1481 (16)	0.082 (17)*	
H7B	−0.019 (2)	0.932 (5)	0.1575 (13)	0.055 (13)*	
H3A	0.383 (3)	0.381 (4)	0.1980 (18)	0.031 (9)*	
H3B	0.501 (4)	0.415 (5)	0.196 (2)	0.070 (15)*	
C1	0.2610 (3)	0.1725 (5)	0.02465 (17)	0.0410 (8)	
H1	0.2129	0.1261	0.0586	0.049*	
C2	0.2030 (3)	0.2200 (5)	−0.03429 (18)	0.0466 (9)	
H2	0.1177	0.2057	−0.0403	0.056*	
C3	0.2754 (4)	0.2894 (5)	−0.08420 (18)	0.0510 (10)	
H3	0.2390	0.3233	−0.1245	0.061*	
C4	0.4011 (4)	0.3084 (5)	−0.07414 (16)	0.0419 (8)	
H4	0.4504	0.3555	−0.1074	0.050*	
C5	0.4537 (3)	0.2565 (4)	−0.01396 (15)	0.0320 (7)	
C6	0.5883 (3)	0.2680 (4)	0.00209 (14)	0.0307 (7)	
C7	0.6786 (3)	0.3242 (4)	−0.04174 (17)	0.0409 (8)	
H7	0.6562	0.3641	−0.0841	0.049*	
C8	0.8013 (4)	0.3203 (5)	−0.02201 (19)	0.0497 (10)	
H8	0.8629	0.3568	−0.0509	0.060*	
C9	0.8320 (3)	0.2617 (5)	0.04121 (19)	0.0501 (10)	
H9	0.9147	0.2564	0.0553	0.060*	
C10	0.7384 (3)	0.2114 (5)	0.08301 (17)	0.0427 (8)	
H10	0.7593	0.1757	0.1261	0.051*	
C11	0.3250 (3)	−0.0638 (4)	0.21041 (16)	0.0341 (7)	
C12	0.4265 (3)	−0.0215 (6)	0.26271 (17)	0.0460 (9)	
H12	0.4210	0.1077	0.2703	0.055*	
C13	0.5590 (3)	−0.0537 (5)	0.23662 (17)	0.0365 (8)	
C14	0.4053 (4)	−0.1058 (6)	0.32924 (19)	0.0652 (12)	
H14A	0.3256	−0.0697	0.3454	0.098*	
H14B	0.4692	−0.0679	0.3600	0.098*	
H14C	0.4075	−0.2336	0.3249	0.098*	
N1	0.3835 (2)	0.1903 (3)	0.03522 (13)	0.0314 (6)	
N2	0.6195 (2)	0.2117 (4)	0.06441 (13)	0.0316 (6)	
O1	0.3381 (2)	−0.0024 (3)	0.15174 (10)	0.0405 (6)	
O2	0.58911 (19)	0.0276 (3)	0.18425 (11)	0.0406 (6)	
O3	0.4394 (3)	0.3907 (3)	0.17161 (12)	0.0388 (6)	
O4	0.2303 (2)	−0.1436 (4)	0.22666 (13)	0.0523 (7)	
O5	0.6349 (2)	−0.1442 (3)	0.26946 (14)	0.0545 (7)	
Cu1	0.47703 (3)	0.12714 (5)	0.118940 (18)	0.03071 (14)	
O6	0.8720 (2)	0.9825 (4)	0.24144 (17)	0.0608 (8)	
O7	0.0314 (3)	0.9009 (6)	0.12845 (16)	0.0828 (11)	

### Atomic displacement parameters (Å^2^)

**Table:**

	*U*^11^	*U*^22^	*U*^33^	*U*^12^	*U*^13^	*U*^23^
C1	0.046 (2)	0.045 (2)	0.0325 (18)	−0.0036 (17)	−0.0021 (16)	0.0004 (16)
C2	0.045 (2)	0.052 (2)	0.042 (2)	0.0005 (18)	−0.0119 (17)	−0.0050 (18)
C3	0.064 (3)	0.052 (2)	0.036 (2)	0.010 (2)	−0.0168 (19)	−0.0014 (18)
C4	0.059 (2)	0.0407 (19)	0.0263 (17)	0.0000 (18)	0.0008 (16)	0.0022 (15)
C5	0.0464 (18)	0.0261 (16)	0.0236 (15)	0.0001 (14)	0.0023 (14)	−0.0029 (13)
C6	0.0469 (19)	0.0223 (15)	0.0229 (15)	−0.0016 (14)	0.0024 (14)	−0.0028 (13)
C7	0.055 (2)	0.0394 (19)	0.0282 (18)	−0.0061 (17)	0.0058 (16)	0.0022 (15)
C8	0.052 (2)	0.054 (2)	0.043 (2)	−0.0116 (19)	0.0184 (19)	−0.0014 (19)
C9	0.0398 (19)	0.063 (3)	0.048 (2)	−0.0080 (19)	0.0047 (17)	0.002 (2)
C10	0.0431 (19)	0.052 (2)	0.0329 (19)	−0.0039 (17)	−0.0006 (16)	0.0004 (17)
C11	0.0293 (16)	0.0436 (19)	0.0294 (17)	0.0002 (14)	0.0034 (13)	0.0054 (15)
C12	0.0388 (19)	0.065 (2)	0.0337 (19)	−0.0006 (18)	0.0018 (16)	0.0082 (18)
C13	0.0286 (16)	0.0427 (19)	0.0381 (19)	−0.0020 (15)	−0.0024 (14)	0.0028 (16)
C14	0.047 (2)	0.109 (4)	0.039 (2)	0.006 (2)	0.0061 (19)	0.004 (2)
N1	0.0349 (14)	0.0313 (13)	0.0280 (14)	−0.0010 (12)	−0.0007 (12)	−0.0020 (12)
N2	0.0322 (13)	0.0357 (15)	0.0271 (14)	−0.0014 (12)	0.0021 (11)	0.0000 (12)
O1	0.0350 (12)	0.0594 (15)	0.0270 (12)	−0.0117 (11)	−0.0024 (10)	0.0077 (11)
O2	0.0300 (11)	0.0548 (15)	0.0372 (13)	0.0014 (11)	0.0044 (10)	0.0168 (12)
O3	0.0406 (14)	0.0477 (15)	0.0283 (13)	0.0007 (12)	0.0040 (12)	−0.0040 (11)
O4	0.0416 (14)	0.0750 (19)	0.0404 (15)	−0.0188 (13)	0.0052 (12)	0.0123 (13)
O5	0.0387 (13)	0.0645 (17)	0.0599 (18)	0.0040 (12)	−0.0082 (13)	0.0288 (14)
Cu1	0.0315 (2)	0.0373 (3)	0.0233 (2)	−0.00150 (17)	0.00136 (15)	0.00288 (16)
O6	0.0354 (15)	0.061 (2)	0.085 (2)	0.0009 (14)	−0.0080 (15)	0.0136 (18)
O7	0.060 (2)	0.132 (3)	0.056 (2)	0.017 (2)	−0.0158 (18)	−0.026 (2)

### Geometric parameters (Å, º)

**Table:**

C1—N1	1.338 (4)	C11—O1	1.276 (4)
C1—C2	1.377 (5)	C11—C12	1.537 (5)
C1—H1	0.9300	C12—C14	1.499 (5)
C2—C3	1.381 (5)	C12—C13	1.545 (4)
C2—H2	0.9300	C12—H12	0.9800
C3—C4	1.372 (5)	C13—O5	1.242 (4)
C3—H3	0.9300	C13—O2	1.263 (4)
C4—C5	1.383 (4)	C14—H14A	0.9600
C4—H4	0.9300	C14—H14B	0.9600
C5—N1	1.348 (4)	C14—H14C	0.9600
C5—C6	1.481 (4)	N1—Cu1	2.004 (3)
C6—N2	1.359 (4)	N2—Cu1	2.001 (2)
C6—C7	1.387 (4)	O1—Cu1	1.907 (2)
C7—C8	1.373 (5)	O2—Cu1	1.919 (2)
C7—H7	0.9300	O3—Cu1	2.276 (2)
C8—C9	1.379 (5)	O3—H3A	0.82 (3)
C8—H8	0.9300	O3—H3B	0.85 (5)
C9—C10	1.374 (5)	O6—H6A	0.836 (10)
C9—H9	0.9300	O6—H6B	0.835 (10)
C10—N2	1.328 (4)	O7—H7A	0.829 (10)
C10—H10	0.9300	O7—H7B	0.839 (10)
C11—O4	1.229 (4)		
			
N1—C1—C2	122.9 (3)	C14—C12—H12	105.3
N1—C1—H1	118.6	C11—C12—H12	105.3
C2—C1—H1	118.6	C13—C12—H12	105.3
C1—C2—C3	118.0 (3)	O5—C13—O2	122.0 (3)
C1—C2—H2	121.0	O5—C13—C12	120.4 (3)
C3—C2—H2	121.0	O2—C13—C12	117.3 (3)
C4—C3—C2	119.9 (3)	C12—C14—H14A	109.5
C4—C3—H3	120.1	C12—C14—H14B	109.5
C2—C3—H3	120.1	H14A—C14—H14B	109.5
C3—C4—C5	119.3 (3)	C12—C14—H14C	109.5
C3—C4—H4	120.4	H14A—C14—H14C	109.5
C5—C4—H4	120.4	H14B—C14—H14C	109.5
N1—C5—C4	121.2 (3)	C1—N1—C5	118.8 (3)
N1—C5—C6	114.8 (3)	C1—N1—Cu1	126.2 (2)
C4—C5—C6	124.0 (3)	C5—N1—Cu1	115.0 (2)
N2—C6—C7	121.0 (3)	C10—N2—C6	118.9 (3)
N2—C6—C5	114.1 (3)	C10—N2—Cu1	126.1 (2)
C7—C6—C5	124.8 (3)	C6—N2—Cu1	115.0 (2)
C8—C7—C6	119.3 (3)	C11—O1—Cu1	126.9 (2)
C8—C7—H7	120.3	C13—O2—Cu1	126.2 (2)
C6—C7—H7	120.3	Cu1—O3—H3A	112 (2)
C7—C8—C9	119.2 (3)	Cu1—O3—H3B	108 (3)
C7—C8—H8	120.4	H3A—O3—H3B	103 (4)
C9—C8—H8	120.4	O1—Cu1—O2	93.10 (9)
C10—C9—C8	118.9 (4)	O1—Cu1—N2	163.97 (10)
C10—C9—H9	120.6	O2—Cu1—N2	91.09 (10)
C8—C9—H9	120.6	O1—Cu1—N1	91.37 (10)
N2—C10—C9	122.7 (3)	O2—Cu1—N1	165.47 (10)
N2—C10—H10	118.7	N2—Cu1—N1	81.04 (10)
C9—C10—H10	118.7	O1—Cu1—O3	97.61 (10)
O4—C11—O1	121.7 (3)	O2—Cu1—O3	97.57 (10)
O4—C11—C12	120.1 (3)	N2—Cu1—O3	97.17 (10)
O1—C11—C12	118.1 (3)	N1—Cu1—O3	95.52 (10)
C14—C12—C11	114.0 (3)	H6A—O6—H6B	105.1 (16)
C14—C12—C13	113.1 (3)	H7A—O7—H7B	104.6 (16)
C11—C12—C13	112.7 (3)		
			
N1—C1—C2—C3	−0.3 (5)	C5—C6—N2—C10	178.0 (3)
C1—C2—C3—C4	0.3 (6)	C7—C6—N2—Cu1	−177.7 (2)
C2—C3—C4—C5	0.3 (6)	C5—C6—N2—Cu1	0.1 (3)
C3—C4—C5—N1	−0.9 (5)	O4—C11—O1—Cu1	178.9 (2)
C3—C4—C5—C6	179.0 (3)	C12—C11—O1—Cu1	4.1 (4)
N1—C5—C6—N2	−0.8 (4)	O5—C13—O2—Cu1	165.5 (3)
C4—C5—C6—N2	179.3 (3)	C12—C13—O2—Cu1	−20.5 (4)
N1—C5—C6—C7	177.0 (3)	C11—O1—Cu1—O2	24.3 (3)
C4—C5—C6—C7	−3.0 (5)	C11—O1—Cu1—N2	129.2 (4)
N2—C6—C7—C8	0.9 (5)	C11—O1—Cu1—N1	−169.5 (3)
C5—C6—C7—C8	−176.7 (3)	C11—O1—Cu1—O3	−73.8 (3)
C6—C7—C8—C9	−0.4 (5)	C13—O2—Cu1—O1	−15.2 (3)
C7—C8—C9—C10	−1.0 (6)	C13—O2—Cu1—N2	−179.7 (3)
C8—C9—C10—N2	2.1 (6)	C13—O2—Cu1—N1	−122.9 (4)
O4—C11—C12—C14	7.4 (5)	C13—O2—Cu1—O3	82.9 (3)
O1—C11—C12—C14	−177.6 (3)	C10—N2—Cu1—O1	−114.8 (4)
O4—C11—C12—C13	138.1 (3)	C6—N2—Cu1—O1	62.9 (5)
O1—C11—C12—C13	−47.0 (4)	C10—N2—Cu1—O2	−9.6 (3)
C14—C12—C13—O5	1.2 (5)	C6—N2—Cu1—O2	168.1 (2)
C11—C12—C13—O5	−129.9 (4)	C10—N2—Cu1—N1	−177.3 (3)
C14—C12—C13—O2	−172.9 (3)	C6—N2—Cu1—N1	0.4 (2)
C11—C12—C13—O2	56.0 (4)	C10—N2—Cu1—O3	88.2 (3)
C2—C1—N1—C5	−0.4 (5)	C6—N2—Cu1—O3	−94.1 (2)
C2—C1—N1—Cu1	179.6 (3)	C1—N1—Cu1—O1	13.4 (3)
C4—C5—N1—C1	1.0 (5)	C5—N1—Cu1—O1	−166.6 (2)
C6—C5—N1—C1	−178.9 (3)	C1—N1—Cu1—O2	121.4 (4)
C4—C5—N1—Cu1	−179.0 (2)	C5—N1—Cu1—O2	−58.7 (5)
C6—C5—N1—Cu1	1.1 (3)	C1—N1—Cu1—N2	179.2 (3)
C9—C10—N2—C6	−1.6 (5)	C5—N1—Cu1—N2	−0.8 (2)
C9—C10—N2—Cu1	176.0 (3)	C1—N1—Cu1—O3	−84.4 (3)
C7—C6—N2—C10	0.1 (5)	C5—N1—Cu1—O3	95.6 (2)

### Hydrogen-bond geometry (Å, º)

**Table:**

*D*—H···*A*	*D*—H	H···*A*	*D*···*A*	*D*—H···*A*
C4—H4···O3^i^	0.93	2.59	3.457 (4)	155
O3—H3*A*···O4^ii^	0.82 (3)	1.97 (4)	2.775 (3)	170 (3)
O3—H3*B*···O6^iii^	0.85 (5)	1.90 (5)	2.744 (4)	173 (4)
O6—H6*A*···O5^iv^	0.84 (2)	1.96 (2)	2.787 (3)	168 (4)
O6—H6*B*···O5^v^	0.84 (1)	1.97 (1)	2.800 (4)	170 (4)
O7—H7*A*···O4^iv^	0.83 (1)	2.12 (2)	2.907 (4)	158 (4)
O7—H7*B*···O6^vi^	0.84 (1)	2.10 (1)	2.932 (5)	170 (4)
C2—H2···O7^vii^	0.93	2.50	3.256 (5)	139
C12—H12···O4^ii^	0.98	2.47	3.300 (5)	142

Symmetry codes: (i) −*x*+1, −*y*+1, −*z*; (ii) −*x*+1/2, *y*+1/2, −*z*+1/2; (iii) −*x*+3/2, *y*−1/2, −*z*+1/2; (iv) *x*, *y*+1, *z*; (v) −*x*+3/2, *y*+3/2, −*z*+1/2; (vi) *x*−1, *y*, *z*; (vii) −*x*, −*y*+1, −*z*.

## References

[bb1] Braga, D., Grepioni, F. & Desiraju, G. R. (1998). *Chem. Rev.* **98**, 1375–1406. 10.1021/cr960091b 11848937

[bb2] Cremer, D. & Pople, J. A. (1975). *J. Am. Chem. Soc.* **97**, 1354–1358.

[bb3] Cui, G.-H., Li, J.-R., Hu, T.-L. & Bu, X.-H. (2005). *J. Mol. Struct.* **738**, 183–187.

[bb4] Farrugia, L. J. (1997). *J. Appl. Cryst.* **30**, 565.

[bb5] Gasque, L., Moreno-Esparza, R., Mollins, E., Briansó-Penalva, J. L., Ruiz-Ramírez, L. & Medina-Dickinson, G. (1998). *Acta Cryst.* C**54**, 1848–1850.

[bb6] Oxford Diffraction (2009). *CrysAlis CCD*, *CrysAlis RED* and *CrysAlis PRO* Oxford Diffraction Ltd, Yarnton, Oxfordshire, England.

[bb7] Sheldrick, G. M. (2008). *Acta Cryst.* A**64**, 112–122. 10.1107/S0108767307043930 18156677

[bb8] Spek, A. L. (2009). *Acta Cryst.* D**65**, 148–155. 10.1107/S090744490804362X PMC2631630 19171970

[bb9] Suresh, E. & Bhadbhade, M. M. (1997). *Acta Cryst.* C**53**, 193–195.

